# The Stem Cell Expression Profile of Odontogenic Tumors and Cysts: A Systematic Review and Meta-Analysis

**DOI:** 10.3390/genes14091735

**Published:** 2023-08-30

**Authors:** Eleni-Marina Kalogirou, Georgios Lekakis, Aristodimos Petroulias, Konstantinos Chavdoulas, Vasileios L. Zogopoulos, Ioannis Michalopoulos, Konstantinos I. Tosios

**Affiliations:** 1 Faculty of Health and Rehabilitation Sciences, Metropolitan College, 10672 Athens, Greece; 2 School of Dentistry, National and Kapodistrian University of Athens, 11527 Athens, Greece; georgelekakis7@gmail.com (G.L.); aristodimospetroulias@gmail.com (A.P.); kostaschvd@gmail.com (K.C.); ktosios@dent.uoa.gr (K.I.T.); 3 Centre of Systems Biology, Biomedical Research Foundation, Academy of Athens, 11527 Athens, Greece; vzogopoulos@bioacademy.gr (V.L.Z.); imichalop@bioacademy.gr (I.M.)

**Keywords:** odontogenic tumor, odontogenic cyst, stem cell, gene expression, marker, ameloblastoma, odontogenic keratocyst, ameloblastic carcinoma, SOX2, systematic review

## Abstract

Background: Stem cells have been associated with self-renewing and plasticity and have been investigated in various odontogenic lesions in association with their pathogenesis and biological behavior. We aim to provide a systematic review of stem cell markers’ expression in odontogenic tumors and cysts. Methods: The literature was searched through the MEDLINE/PubMed, EMBASE via OVID, Web of Science, and CINHAL via EBSCO databases for original studies evaluating stem cell markers’ expression in different odontogenic tumors/cysts, or an odontogenic disease group and a control group. The studies’ risk of bias (RoB) was assessed via a Joanna Briggs Institute Critical Appraisal Tool. Meta-analysis was conducted for markers evaluated in the same pair of odontogenic tumors/cysts in at least two studies. Results: 29 studies reported the expression of stem cell markers, e.g., SOX2, OCT4, NANOG, CD44, ALDH1, BMI1, and CD105, in various odontogenic lesions, through immunohistochemistry/immunofluorescence, polymerase chain reaction, flow cytometry, microarrays, and RNA-sequencing. Low, moderate, and high RoBs were observed in seven, nine, and thirteen studies, respectively. Meta-analysis revealed a remarkable discriminative ability of SOX2 for ameloblastic carcinomas or odontogenic keratocysts over ameloblastomas. Conclusion: Stem cells might be linked to the pathogenesis and clinical behavior of odontogenic pathologies and represent a potential target for future individualized therapies.

## 1. Introduction

Odontogenic tumors and cysts are a diverse group of lesions that have in common their origin from cells participating in the normal process of tooth formation or odontogenesis. Benign and malignant odontogenic tumors are rare, representing less than 1% of oral tumors [1], and present a wide variety of clinical behavior and histopathological features. Some of them behave in a destructive manner, while others are slow-growing and may be even accidentally discovered during the microscopic examination of the follicular tissue surrounding an unerupted third molar (dental follicle, DF) [2].

The most common benign odontogenic tumor is ameloblastoma (AMBL), a locally infiltrative neoplasm with a high recurrence rate that may occasionally undergo malignant transformation [1]. AMBL often necessitates wide surgical excision, associated with high morbidity and necessitating extensive reconstructive surgery; thus, pharmaceutical-based management is a challenging treatment goal [3]. In contrast to AMBL, adenomatoid odontogenic tumor (AOT), another benign odontogenic tumor, has limited growth potential and significantly lower recurrence rate [1].

Odontogenic cysts are of developmental and inflammatory origin. The most common developmental odontogenic cyst is the dentigerous cyst (DC), which is associated with the crown of unerupted teeth, grows slowly, and rarely recurs [1,4]. Odontogenic keratocyst (OKC) is a developmental odontogenic cyst with great research and clinical interest, due to its high frequency and aggressive behavior, i.e., growth potential within jaw bones and high recurrence rate, as well as its occurrence as a manifestation of Gorlin–Goltz syndrome [1]. Radicular cyst (RC) is the most common among all odontogenic cysts and develops within the jawbones as a sequel of dental pulp necrosis [1,4].

The odontogenic tissues develop through constant epithelial–mesenchymal interactions, where stem cells play a pivotal role [5]. Odontogenic tumors and cysts purportedly arise from cells of the odontogenic tissues and their developmental remnants, such as dental lamina and epithelial rests of Malassez, where stem cells have been shown to exist [6,7]. Stem cells have been associated with self-renewing and plasticity, thus contributing to different organs’ formation and regeneration [5], while, in cases of disturbed and uncontrolled proliferation, stem cells might promote tumorigenesis [8]. Moreover, based on their ability to differentiate various specialized subpopulations, stem cells have been linked to morphological heterogeneity and diverse biological behavior [9], features that characterize odontogenic pathologies.

Previous studies have focused on the expression of a limited number of stem cell markers in odontogenic tumors and cysts [10,11,12,13,14]; however, the complete stem cell gene expression portrait of odontogenic lesions remains elusive. Understanding the role of stem cells in odontogenic lesions can contribute to the development of personalized study models (“disease-in-a-dish” models), which are of great importance for the study of the pathogenesis of rare diseases [15]; identification of stem cell genes that might be targeted for personalized molecular treatments [16], in particular for the management of large osteolytic lesions; and the utilization of stem cells in regenerative techniques in the oral and maxillofacial region [17].

Herein, we aim to provide a thorough systematic review of studies investigating the expression of stem cell markers in odontogenic tumors and cysts. The null hypothesis is that there are no significant differences in the expression of stem cell markers between various odontogenic lesions.

## 2. Materials and Methods

### 2.1. Research Question and Study Protocol

The research question of the study was formulated using the Population, Intervention, Comparator, and Outcome framework as follows: “Are there any differences in the expression of stem cell markers between various odontogenic lesions?” (Population = odontogenic tumors and cysts, Intervention = not applicable, Comparator = stem cell markers, Outcome = differences in expression). The Preferred Reporting Items for Systematic Reviews and Meta-Analyses (PRISMA) guidelines [18] were applied for the study implementation, and the study protocol was registered in the database of the Prospective International Registration of Systematic Reviews [19] and received the reference number CRD42023415311.

### 2.2. Search Strategy

**Figure 1:**
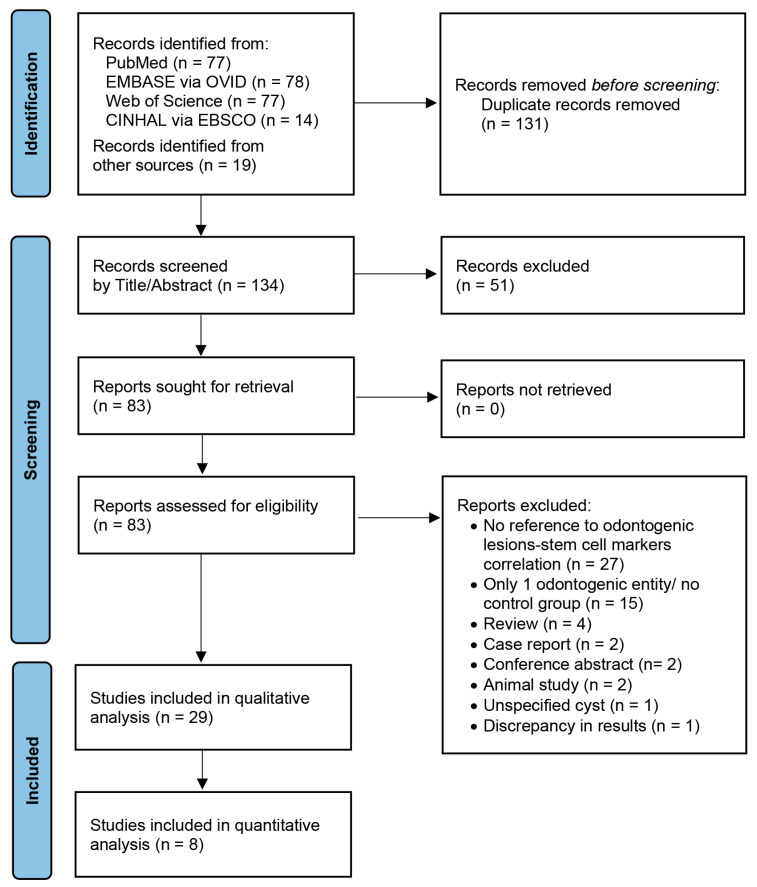
The PRISMA [18] flow diagram illustrating the search strategy.

The literature search was performed on 10 April 2023 through the MEDLINE/PubMed, EMBASE via OVID, Web of Science, and CINHAL via EBSCO host databases to identify articles published in peer-reviewed journals and written in the English language, which included the following keywords in the title or abstract: (ameloblastoma* or adenomatoid odontogenic tumo* or squamous odontogenic tumo* or calcifying epithelial odontogenic tumo* or Pindborg tumo* or primordial odontogenic tumo* or ameloblastic fibroma* or dentinogenic ghost tumo* or odontogenic fibroma* or odontoma* or cementoblastoma* or cemento-ossifying fibroma* or odontogenic myxoma* or sclerosing odontogenic carcinoma* or ameloblastic carcinoma* or clear cell odontogenic carcinoma* or primary intraosseous carcinoma* or ghost cell odontogenic carcinoma* or odontogenic carcinosarcoma* or odontogenic sarcoma* or odontogenic tumo* or odontogenic neoplas* or radicular cyst* or inflammatory collateral cyst* or surgical ciliated cyst* or nasopalatine duct cyst* or gingival cyst* or dentigerous cyst* or orthokeratinised odontogenic cyst* or lateral periodontal cyst* or botryoid odontogenic cyst* or calcifying odontogenic cyst* or Gorlin cyst* or calcifying cystic odontogenic tumor* or glandular odontogenic cyst* or odontogenic keratocyst* or keratocystic odontogenic tumo* or odontogenic cyst*) and (stem cell* or pluripoten*). The search terms in each database are presented in Table S1. The reference lists of retrieved articles were manually browsed for relevant studies. At this stage, studies in languages other than English were excluded (Table S2). Records were imported to Endnote X8 citation manager, and duplicates were automatically removed (Figure 1).

### 2.3. Eligibility Screening and Study Selection

The titles and abstracts of the retrieved studies were screened by three researchers (G.L., A.P., and K.C.) independently to identify original studies investigating the expression of stem cell gene markers in human benign or malignant odontogenic tumors, as well as odontogenic cysts of developmental or inflammatory origin (Figure 1). Case reports/short case studies/letters to the editor/correspondence/commentaries without any stem cell expression data, narrative/systematic reviews, conference abstracts, and animal studies were excluded at this phase (Table S2). In cases of questionable suitability by title/abstract, the full text was downloaded.

The full text of studies considered as suitable at previous search steps were investigated for eligibility by three researchers (G.L., A.P., and K.C.) independently and any cases with discrepancy were resolved by asking for an opinion from the other two authors (E.M.K. and K.I.T.) (Figure 1). Eligibility criteria were: (1) original studies investigating the expression of core stem cell gene markers [20,21,22] in human fresh-frozen or formalin-fixed, paraffin-embedded (FFPE) tissues of odontogenic tumor(s)/cyst(s), or in human odontogenic tumor/cyst-derived cell lines, and (2) original studies including at least two different odontogenic tumor/cyst entities, one odontogenic tumor group and one odontogenic cyst group, or one odontogenic tumor/cyst group and a normal odontogenic control group.

In articles including multiple gene markers or multiple methods, the eligibility criteria were applied on each marker/method individually. Genes known to be surface markers of embryonic stem cells (ESCs) or cancer stem cell biomarkers, with a principal role in stem cell phenotype induction and/or maintenance [20,21,22], were regarded as eligible markers when their expression was evaluated in major components of odontogenic lesions, i.e., the epithelium/parenchyma or the supporting stroma, but not when they were assessed as markers of specific stromal structures (e.g., vessels). Eligible methods were those applied to assess the stem cell markers’ expression in different odontogenic groups, either supported by statistical analysis or not.

Studies excluded from qualitative analysis at any search phase and the reasons for exclusion are presented in Table S2.

### 2.4. Data Extraction and Synthesis

Four authors (E.M.K., G.L., A.P., and K.C.) extracted the following data in Microsoft Office Excel 365 spreadsheets:

(1) Data regarding the reference, i.e., authorship, publication year, and journal, and the study population, i.e., sample type, number of patients, number of samples in each odontogenic lesion/control group, demographic characteristics, location of odontogenic lesions/control samples, whether a detailed histopathologic description or a reference (e.g., World Health Organization edition) was provided to support the diagnosis of the included odontogenic entities, and the histopathological variants in case of solid ameloblastomas (AMBLs) (Table S3, sorted by the most recent article).

(2) Information about the method applied for the evaluation of stem cell marker(s)’ expression and the stem cell marker(s) as recorded by the protein/gene name provided by each publication and by the Human Genome Organization (HUGO) Gene Nomenclature [23] (Table S4, sorted by the protein-coding gene name in alphabetical order). In addition, in case of immunohistochemistry/immunofluorescence-based studies on tissues, information about the antibodies and technique (i.e., host, clonality, dilution catalogue name/number, source, tissue section thickness, and positive and negative control tissues), as well as the evaluation method, the scoring system, and the number of staining observers were recorded (Table S4). Methodological details of studies employing flow cytometry analysis, (i.e., antibody, fluorochrome, and analysis instrument), polymerase chain reaction (PCR) experiments (i.e., type of PCR, input DNA/RNA size, primers’ sequence, and housekeeping gene) or gene expression profiling methods, i.e., microarrays and RNA-sequencing (RNA-Seq), are summarized in Table S5 (sorted by the type of method).

(3) Information about the expression results, i.e., number or percentage of positive cases and/or expression score, in case of studies on tissues, or percentage of positive cells in case of in vitro experiments, as well as the results of differential expression analysis (Table S6, sorted by the protein-coding gene name in alphabetical order, and Table S7, sorted by the type of method). For studies reporting the immunolocalization of stem cell markers, additional descriptive information was recorded, such as the tissue (epithelial/stromal expression), the compartmental (peripheral/central cells for tumor epithelial islands or basal/suprabasal/superficial layer for cystic epithelium) and/or the subcellular (nuclear/cytoplasmic/membranous expression) localization (Table S6). Finally, the studies’ conclusions about the comparison of stem cell markers’ expression between different odontogenic tumor/cyst groups or between the odontogenic lesion group(s) and the control group, supported or not by statistical analysis, were recorded (Tables S6 and S7).

### 2.5. Risk of Bias Assessment

Quality assessment was performed by two investigators (G.L. and A.P.) independently, and any disagreements were resolved through discussion with a third author (E.M.K.). Based on the retrospective nature and small sample size of the eligible articles that did not include follow-up data, the risk of bias (RoB) was estimated with the Critical Appraisal Tool, proposed by the Joanna Briggs Institute (JBI) [24], applied in our previous systematic review on immunohistochemical studies [25] and further modified to also be applicable to other methods (Table S8). This JBI RoB tool encompasses 10 items that were marked as “Yes” (low risk), “No” (high risk), “Unclear” (moderate/unclear risk), or “Not applicable” in each study [24]. For studies with multiple gene markers or methods, each question was marked considering the answers for all gene markers/methods, and the total risk of bias was reported. A question was marked as “Yes” only if “Yes” applied to all markers, while it was marked as “No” if at least one marker was scored with “No”. Accordingly, if the required information for a “Yes” mark in one item was provided for some, but not all, methods, the final mark for that item was “Unclear”. The final RoB score was calculated based on the percentage of “Yes” marks, i.e., a “Yes” rate of up to 49%, 50–69%, or ≥70%, corresponding to high, moderate, or low RoB, respectively [25]. Two figures depicting the risk of bias for every item in each study and the summary of the risk of bias per item were generated with Review Manager 5.4 [26].

### 2.6. Meta-Analysis

Aiming to evaluate the discriminative ability of stem cell gene markers between different odontogenic entities, a meta-analysis was performed for markers whose expression was tested (a) between the same pair of odontogenic entities, (b) in at least two studies, and (c) was evaluated in the same microscopic compartment (epithelium or stroma). Meta-analysis was conducted on the studies that passed the selection criteria, using Meta-DiSc Version 1.4 [27], as previously described [25].

## 3. Results

### 3.1. Study Cohort

The systematic literature search yielded 246 records through four electronic databases, and 19 records manually (Figure 1). After 131 duplicates were removed, the title and abstract of 134 studies were screened. Fifty-one articles were eliminated at this step, due to non-English language, i.e., Chinese, Russian, or Spanish; type of study, i.e., case report, review, or conference abstract; or because they did not meet the study aim or the eligibility criteria. For a total of 83 studies, the full text was evaluated, and 54 articles were considered as not eligible and were excluded at this phase, as they were out of the study scope; included only one odontogenic entity, without a control group; were not original studies; or presented unclear data (Table S2). Finally, the qualitative analysis included twenty-nine studies (Table 1), while eight studies [12,14,28,29,30,31,32,33] provided sufficient data for quantitative analysis (meta-analysis). The 29 eligible studies had been issued in 25 different scientific journals (Table S3) during the years 2000–2023, with 24 out of 29 studies being published after 2010.

### 3.2. Study Population Characteristics

Various combinations of benign and malignant odontogenic tumors, and odontogenic cysts with developmental or inflammatory origin, alone or compared with non-disease/lesional controls, constituted the study population (Table 1). In 12 of 29 studies, the expression of stem cell markers was evaluated in parallel in odontogenic tumors and odontogenic cysts, either also including a non-lesional group (three out of twelve) or not (nine out of twelve). Nine out of twenty-nine studies included only odontogenic tumors, either in comparison to a normal control group (six out of nine) or not (three out of nine). Eight out of twenty-nine studies involved only odontogenic cysts, compared with a control group (five out of eight) or not (three out of eight). As seen in Table 1, the most encountered odontogenic lesion was a benign odontogenic tumor, i.e., AMBL, that comprised one of the study groups in 20 of 29 studies, followed by two developmental odontogenic cysts, i.e., OKC (13 out of 29) and DC (10 out of 29). In 13 of 20 studies with AMBLs, the histopathologic variant was specified, with the follicular variant being the most frequent (Table S3). Eight studies included samples of inflammatory odontogenic cysts, i.e., RC, while cases of malignant odontogenic tumors, e.g., ameloblastic carcinoma (ACA), malignant AMBL, metastasizing AMBL, clear cell odontogenic carcinoma or ameloblastic fibrosarcoma, were encompassed in the study groups of five studies (Table 1). In 12 of 29 studies, a non-lesional control group existed, usually DFs. When RCs represented the only disease group, periapical granulomas were selected as controls (Table 1).

The exact number of patients who contributed the study samples was available in 15 out of 29 articles (Table S3). Demographic information was available for all or some of patients in eleven and five studies, respectively, while clear data about the maxillary or mandibular location of all study samples were available in eight records (Table S3). The vast majority of studies (24 out of 29) reported experiments on FFPE tissue samples (Table S3), two studies included both fresh-frozen and FFPE samples [6,39], one study reviewed only fresh-frozen samples [42], and in vitro analysis of human odontogenic lesion-derived cells was performed in two studies [43,47]. A minority of studies (10 out of 29) documented the microscopic diagnosis of included odontogenic entities, either by providing a detailed microscopic description [6,11,12,29,35] or by mentioning an appropriate reference [13,32,36,37,39], e.g., the WHO 2017 [1] or 2005 [51] editions for odontogenic tumors and cysts.

### 3.3. Study Comparator Characteristics

Nineteen different stem cell markers were used to investigate the presence of cells with stem cell properties in odontogenic tumors and cysts (Table 1). SOX2 expression was evaluated in fifteen studies, CD44 and OCT4 in eight and six studies, respectively, and each of ALDH1, BMI1, CD73, CD105, and NANOG were assessed in two studies, while the positivity of each one of the remaining markers was assessed in a single study (Table 1).

All but one of the retrieved studies reported the expression of stem cell markers in odontogenic tumors and cysts based on immunohistochemical or immunofluorescence analyses (Table 1). One study used flow cytometry to compare the expression of stem cell markers in cells derived from odontogenic cysts and dental pulp stem cells [43], two studies confirmed the expression of stem cell markers via reverse transcription-PCR (RT-PCR) and immunohistochemistry [13,47], and two studies employed advanced molecular biology methods, i.e., microarrays on fresh-frozen samples [42] and RNA-seq on FFPE tissues [37], and validated their results via RT-PCR and/or immunohistochemistry/immunofluorescence. Tables S4 and S5 present detailed information regarding the immunohistochemistry/immunofluorescence experiments and the other techniques, respectively. Multiple antibodies and/or dilutions for the same markers were tested in immunohistochemical or immunofluorescence experiments of different studies, i.e., anti-SOX2 antibodies from eleven companies, anti-CD44 from six companies, and anti-OCT4 from five companies (Table S4). Leonardi et al. [50] tried one monoclonal and one polyclonal anti-CD44 antibody from the same company. According to 23 out of the 29 studies with available information, the evaluation of immunohistochemical results was performed via a quantitative (six out of twenty-three) or semiquantitative (seventeen out of twenty-three) scoring system on tissue sections of 3–5 μm thickness (thickness reported in 22 of 29 articles) by one, two, three, or six observers (number of observers available in 15 of 29 articles) (Table S4). In three out of four studies that reported RT-PCR experiments, real-time RT-PCR was applied, and the primers’ sequence was available for all evaluated genes (Table S5). Flow-cytometric analysis using six antibodies conjugated with three different fluorochromes was performed in one study [43]. Affymetrix technology was employed in one microarray-based study [42], while Illumina technology was selected for whole transcriptomics analysis in a single study [42].

### 3.4. Outcome of Stem Cell Markers’ Expression

**Figure 2:**
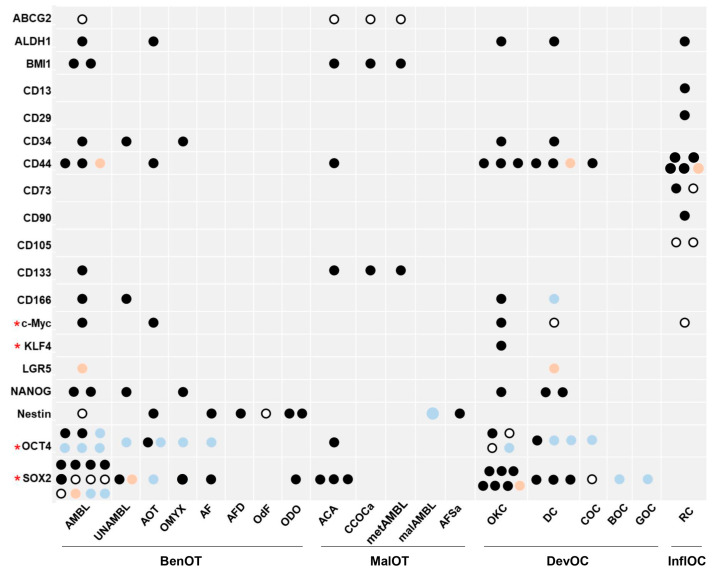
Expression results for each marker in each odontogenic lesion. For each odontogenic lesion, each dot corresponds to the results of one study, as follows: black dot, if the marker was expressed in >50% of cases/cells of this lesion; white dot with black border, if the marker was expressed in up to 50% of cases/cells of this lesion; orange dot, if the marker was expressed in that lesion, but the percentage of positive cases/cells was not available; and light blue dot, if the marker was negative in all cases of that lesion. If the cell is empty, then no study had evaluated the expression of this marker in that odontogenic lesion. Markers indicated with a red * belong to the so called “Yamanaka factors” [52]. Abbreviations: AMBL, ameloblastoma; ACA, ameloblastic carcinoma; AF, ameloblastic fibroma; AFD, ameloblastic fibro-odontoma/fibrodentinoma; AFSa, ameloblastic fi-brosarcoma; AOT, adenomatoid odontogenic tumor; BenOT, benign odontogenic tumor; BOC, botryoid odontogenic cyst; CCOCa, clear cell odontogenic carcinoma; COC, calcifying odontogenic cyst; DC, dentigerous cyst; DevOC, developmental odontogenic cyst; GOC, glandular odontogenic cyst; InflOC, inflammatory odontogenic cyst; malAMBL, malignant AMBL; MalOT, malignant odontogenic tumor; metAMBL, metastasizing AMBL; OdF, odontogenic fibroma; ODO, odontoma; OKC, odontogenic keratocyst; OMYX, odontogenic myxoma; RC, radicular cyst; UNAMBL, unicystic ameloblastoma.

All evaluated stem cell markers were expressed in at least one odontogenic entity, and, according to most studies with available information regarding localization, they were predominantly expressed in the tumor parenchyma or cystic epithelium (Tables S6 and S7). Figure 2 illustrates the expression results for each marker in benign and malignant odontogenic tumors, as well as in developmental and inflammatory odontogenic cysts.

#### 3.4.1. Benign Odontogenic Tumors

AMBL was found to express ABCG2 [13], ALDH1 [34], BMI1 [13,40], CD133 [13], CD166 [41], CD44 [12,31,44], and c-Myc [45] in peripheral and/or central cells of the parenchyma at variable extent and intensity levels, as well as CD34 in spindle-shaped stomal cells [10] and LGR5 [39] and NANOG [10,14] in both epithelial and stromal tumor components. AMBL cases were completely negative for OCT4 in four studies [10,12,28,32], and 100% positive in epithelial cells in two studies [14,31]. Of note, two studies using rabbit anti-OCT4 antibodies from the same company, one monoclonal at 1:200 dilution [10] and one polyclonal at 1:800 dilution [31], demonstrated completely opposite results in AMBL. In seven out of twelve studies assessing SOX2 expression in AMBL, most or all cases were negative (Table S6). In contrast, five studies revealed SOX2 positivity in the majority of peripheral and/or central cells of epithelial islands [6,14,33,38] and/or stomal cells [6,10]. In most studies reporting stem cell marker expression data in different histopathological variants of AMBL, no significant differences were observed between the microscopic variants [6,13,34,41,42,45]. Unicystic AMBL expressed CD166 in the cystic lining [41], CD34 and NANOG in stromal cells [10], and SOX2 in epithelial [38] and stromal cells [10], while it was OCT4 negative [10].

AOT expressed ALDH1 [34], CD44 [31], c-Myc [45], and Nestin [49], mainly in epithelial cells of tumor nests and rosette-like structures. One study found that AOT was 100% positive for OCT4 [31], whereas another study using an anti-OCT4 antibody from a different company resulted in 100% negative results [32]. The latter study also reported an absence of staining for SOX2 in AOT samples [32].

CD34, NANOG, and SOX2, but not OCT4, were expressed in the stromal cells of odontogenic myxoma [10]. Ameloblastic fibroma tissue samples were found to be positive for Nestin [49] and SOX2 [32], and negative for OCT4 [32]. Nestin stained mainly the ectomesenchyme, but also the epithelial islands of ameloblastic fibrodentinoma and the ectomesenchyme of odontogenic fibroma [49], as well as the odontoblasts, pulp cells associated with odontoblasts, cells adhering to dentine in odontoma tissue samples [49], and cells isolated from a human odontoma case [47], which were additionally shown to express the SOX2 protein [47].

#### 3.4.2. Malignant Odontogenic Tumors

Expression of ABCG2, BMI1, and CD133 at the mRNA and protein level was reported in cases of metastasizing AMBL, ACA, and clear cell odontogenic carcinoma [13]. Nestin staining was seen in samples of ameloblastic fibrosarcoma, but not of malignant AMBL [49]. Interestingly, three studies confirmed strong nuclear SOX2 expression in ACA in epithelial areas of cytological atypia, and loss of classic ameloblastic differentiation [12,29,30].

#### 3.4.3. Developmental Odontogenic Cysts

OKC expressed ALDH1 [34], CD166 [41], CD44 [31,46,48], c-Myc [45], and KLF4 [37] in the cystic lining, predominantly in the basal and/or intermediate layers, and CD34 and NANOG in cells of the cystic capsule [10]. OCT4 was negative [28] or focally expressed in the superficial epithelial layer [32] or in a few stromal cells of OKC [10], whereas one study employing a polyclonal anti-OCT4 antibody reported 100% positivity in OKC [31]. In contrast, six studies agreed on the strong nuclear expression of the SOX2 protein in OKC epithelial cells, mainly in the basal and/or intermediate layers [28,32,33,36,37,42], while one study showed SOX2 stromal positivity [10]. In addition, SOX2 RNA was significantly upregulated compared with AMBL or DF, according to a microarray-based [42] and an RNA-Seq-based study [37], respectively.

DC expressed CD44 [44,46,48] and c-Myc [45] in the cystic epithelium; CD34 in the cystic capsule [10]; and ALDH1 [34], NANOG [10,14] and SOX2 [10,32] in epithelial and/or mesenchymal cells. One study reported OCT4 positivity [14], whereas two studies showed no expression of the same marker in DC [10,32].

Calcifying odontogenic cysts were positive for CD44 [46], but negative in all or most cases for OCT4 and SOX2, respectively [32]. Similarly, botryoid odontogenic cysts and glandular odontogenic cysts did not express SOX2 [36].

#### 3.4.4. Inflammatory Odontogenic Cysts

Five immunohistochemical studies found expression of the CD44 protein in either the cystic epithelium [44,46,48] or the inflammatory milieu of the cystic capsule [11], or both compartments [50] of the RC. One of the studies used a monoclonal antibody against the CD44H standard form and a polyclonal antibody against the CD44V3 isoform, with the same results [50]. Immunohistochemical experiments on FFPE tissue samples also revealed staining of c-Myc in the epithelium of RC [45], of CD73 and CD105 in the cystic capsule [11], and of ALDH1 in both epithelial and mesenchymal cells of RC [35]. Moreover, flow cytometric analysis confirmed CD44, CD73, and CD105 expression in mesenchymal cells isolated from human RCs (HRCMCs), and also showed CD13, CD29, and CD90 expression in those cells at similar levels with dental pulp stem cells (DPSCs), except for CD105, which was underexpressed in HRCMCs compared to DPSCs [43]. RT-PCR analysis also revealed low Nestin mRNA levels in HRCMCs, as in DPSCs [43].

### 3.5. RoB Assessment

**Figure 3:**
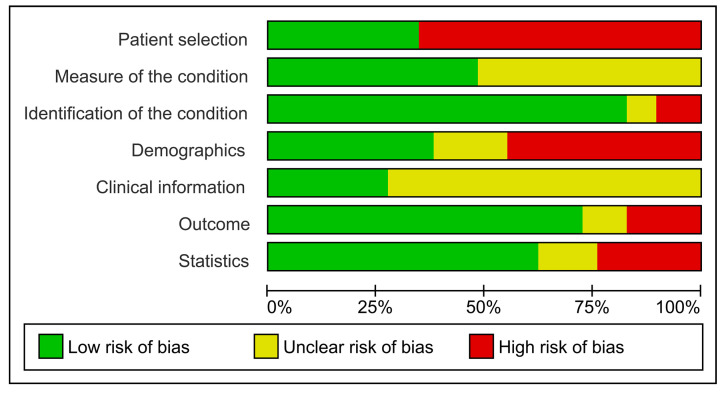
Risk of bias assessment via the Critical Appraisal Tool by Joanna Briggs Institute [24].

Seven, nine, and thirteen studies were characterized by low, moderate (unclear), or high RoB, respectively (Supplementary Table S9), according to the Joanna Briggs Institute Critical Appraisal Tool [24]. All studies with low RoB were published between 2018 and 2023, whereas most studies (nine out of fourteen) issued before 2018 had high RoB (Table S9). Figure 3 summarizes the RoB classification for each item of the applied Critical Appraisal Tool [24]. The diagnostic criteria supporting “Patient selection” were reported only in 10 of 29 (34.5%) studies. Another item with a considerable proportion of unclear or high RoB was “Demographics”, as less than half of studies (11 of 29, 37.9%) provided gender and age information for all study groups. Similarly, the “Clinical information” item showed predominantly an unclear RoB, as only eight of twenty-nine (27.6%) studies adequately reported the number of patients and site of lesions in every study group. The “Measure of the condition” item, corresponding to the detailed description of each study methodology, was of low RoB in 14 of 29 studies (48.3%), as 15 studies were lacking information about dilution and/or positive/negative controls (Tables S4 and S5) and were classified with unclear RoB for this item. A total of 24 out of 29 (82.8%) studies provided details about the evaluation methods and/or scoring systems, resulting in low RoB for the “Identification of the condition” item. Most studies also presented low RoB for items “Outcome” (21/29, 72.4%) and “Statistics” (18/29). In contrast, critical information about the stem cell gene expression, e.g., the number of positive cases and/or the tissue localization of expression, was not mentioned in seven studies, and statistical analysis was missing for some or all methods in eleven studies (Tables S4–S6).

### 3.6. Meta-Analysis

Eight studies met the selection criteria and were included in the quantitative analysis (Table S10). Three, three, and two studies, evaluating the SOX2 expression in the epithelium of ACA vs. AMBL [12,29,30], in the epithelium of OKC vs. AMBL [28,32,33], and the epithelium of AMBL vs. DF [12,14], respectively, were eligible for meta-analysis. Moreover, two studies comparing the OCT4 epithelial expression in OKC vs. AMBL [31,32] were suitable for meta-analysis.

#### 3.6.1. SOX2 in ACA vs. AMBL

**Figure 4:**
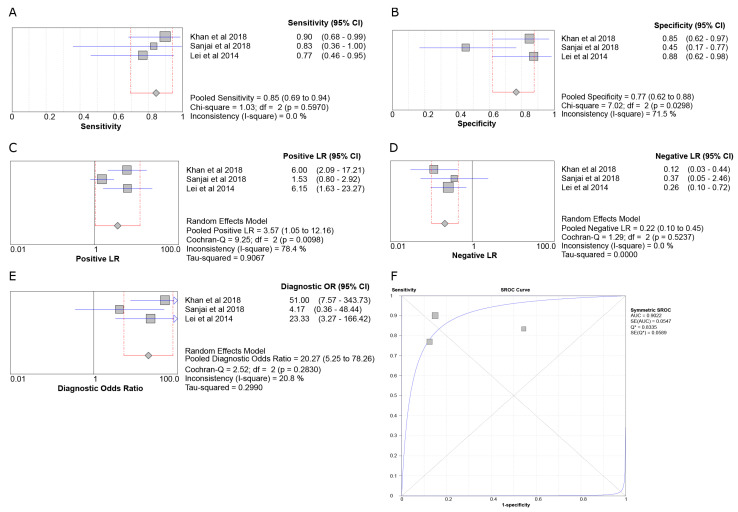
Forest plots of the pooled Sensitivity (**A**), Specificity (**B**), LR+ (**C**), LR- (**D**), DOR (**E**), and SROC curve (**F**) of the three studies [12,29,30] involving the SOX2 marker for the ACA and AMBL disease pair.

In the ACA vs. AMBL meta-analysis for SOX2 expression (Figure 4), Sensitivity was 0.85 (95% CI 0.69–0.94), Specificity was 0.77 (95% CI 0.62–0.88), the Positive Likelihood Ratio (LR+) was 3.57 (95% CI 1.05–12.16), the Negative Likelihood Ratio (LR-) was 0.22 (95% CI 0.10–0.45), and the Diagnostic Odds Ratio (DOR) was 20.27 (95% CI 5.25–78.26). Sensitivity and LR- had no heterogeneity between the studies (I^2^ = 0%), DOR had insignificant heterogeneity (I^2^ = 20.8%), Specificity had moderate heterogeneity with an I^2^ of 71.5%, and LR+ had high heterogeneity with an I^2^ of 78.4%. Finally, the diagnostic usefulness of SOX2 for the differential diagnosis of ACA and AMBL was high (Area Under the Curve (AUC) = 0.9022).

#### 3.6.2. SOX2 in OKC vs. AMBL

**Figure 5:**
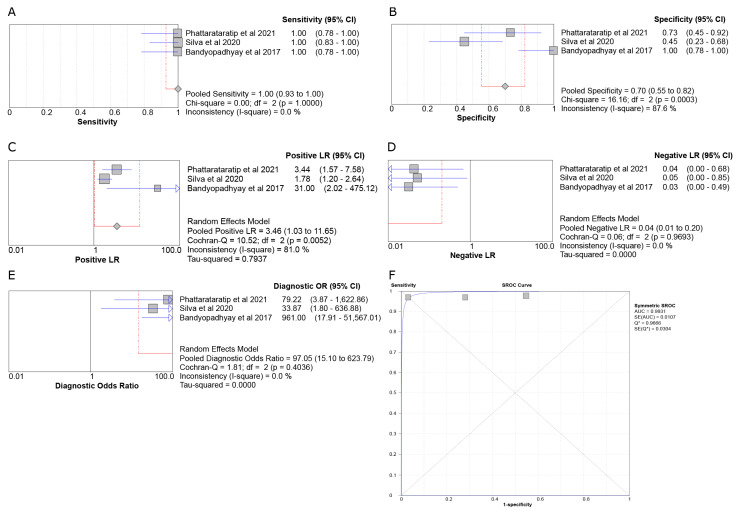
Forest plots of the pooled Sensitivity (**A**), Specificity (**B**), LR+ (**C**), LR- (**D**), DOR (**E**), and SROC curve (**F**) of the three studies [28,32,33] involving the SOX2 marker for the OKC and AMBL disease pair.

In the OKC vs. AMBL meta-analysis for SOX2 expression (Figure 5), Sensitivity was 1.00 (95% CI 0.93–1.00), Specificity was 0.70 (95% CI 0.55–0.82), LR+ was 3.46 (95% CI 1.03–11.65), LR- was 0.04 (95% CI 0.01–0.20), and DOR was 97.05 (95% CI 15.10–623.79). Sensitivity, LR-, and DOR showed no heterogeneity, while Specificity and LR+ presented high heterogeneity with an I^2^ of more than 80%. The discriminative ability of SOX2 between OKC and AMBL was outstanding (AUC = 0.9931).

#### 3.6.3. SOX2 in AMBL vs. DF

In the AMBL vs. DF meta-analysis for SOX2 expression (Figure S1), Sensitivity was 0.60 (95% CI 0.44–0.75), Specificity was 0.23 (95% CI 0.08–0.55), LR+ was 0.78 (95% CI 0.23–2.65), LR- was 0.74 (95% CI 0.09–6.24), and DOR was 1.11 (95% CI 0.04–29.21). LR+, LR-, and DOR presented moderate heterogeneity with I^2^ values of 65.6%, 53.3%, and 64.4%, respectively, while Sensitivity and Specificity had high heterogeneity with I^2^ values of 97.5% and 76%, respectively. Since only two studies were included, no summary Receiver Operating Characteristic (SROC) curve was produced and, thus, AUC was not calculated.

#### 3.6.4. OCT4 in OKC vs. AMBL

In the OKC vs. AMBL meta-analysis for OCT4 expression (Figure S2), Sensitivity was 0.63 (95% CI 0.45–0.79), Specificity was 0.46 (95% CI 0.29–0.63), LR+ was 1.70 (95% CI 0.19–15.61), LR- was 0.87 (95% CI 0.69–1.09), and DOR was 4.31 (95% CI 0.45–41.10). LR- and DOR presented no heterogeneity between the studies (I^2^ = 0%), while LR+ presented moderate heterogeneity (I^2^ = 61.8%). Sensitivity and Specificity had high heterogeneity with I^2^ of values of 97.1% and 97.5%, respectively. Since there were only two studies included, no SROC plot could be produced.

## 4. Discussion

The present study summarizes for the first time the evidence available in the literature on the expression of stem cell gene markers in odontogenic tumors and cysts, and further highlights the usefulness of SOX2 in the differential diagnosis of odontogenic lesions with divergent biological behavior.

Most relevant studies investigated AMBL, OKC, DC, and RC, the first two apparently due to their aggressive biological behavior [1], and the latter two probably due to the wide availability of tissue, as they are the most common odontogenic cysts [4]. In contrast, only a few studies included samples of malignant odontogenic tumors, profoundly due to the rarity of such lesions [1]. In most studies, the expression of stem cell markers was documented via immunohistochemistry and/or immunofluorescence. Although by modern molecular techniques, the expression of tens to hundreds of thousands of genes may be investigated in parallel [53], immunohistochemistry remains the gold-standard for the detection of the tissue-specific expression of proteins and their precise subcellular localization [54]. The latter may be important for unveiling their function, e.g., in case of transcription factors, where nucleo-cytoplasmic shuttling significantly influences their activity [55].

Among the eighteen stem cell markers identified in this systematic review (Table 1), SOX2 was the most studied. The SOX2 protein is encoded by the *SOX2* (SRY-box transcription factor 2) gene that is expressed in ESCs and adult tissue stem cells, and exerts an important role in the development of tissues of ectodermal origin, including the odontogenic epithelium [6,56]. SOX2 is also one of the main four “Yamanaka factors”, i.e., transcription factors whose exogenous administration to differentiated somatic cells can induce their reprogramming into induced pluripotent stem cells (iPSCs), through the process of cellular reprogramming [52]. In addition to its role in the normal development and homeostasis of the covering mucosal epithelium, SOX2 participates in tumorigenesis, affecting the proliferation, apoptosis, and cell differentiation of malignant neoplasms originating from various tissues, such as oral and skin squamous cell carcinomas [57]. According to our meta-analysis, SOX2 has a remarkable ability in identifying cases of ACA over AMBL, its benign counterpart. In ACA, strong nuclear SOX2 expression was observed in areas with prominent cytological atypia and loss of the classical ameloblastic morphology, whereas the few positive AMLB cases showed weak, focal SOX2 staining in peripheral or central cells of epithelial islands and strands [12,29,30]. Those findings suggest that SOX2 immunostaining could facilitate the diagnosis of ambiguous cases of ACA and reveal malignant transformation in AMBL [30].

Furthermore, the quantitative analysis performed in the present study indicated that SOX2 expression between OKC and AMBL is different. This finding is significant as it could be associated with differences in the pathogenesis of those lesions, while diagnostically it could be utilized in the identification of ameloblastic transformation in OKC. Previous studies have shown nuclear expression of SOX2 [6] in the dental lamina, focally in the dental lamina rests included in DFs [37,40], and cytoplasmic expression of SOX2 in ameloblasts, odontoblasts, and inner enamel epithelium cells of human fetuses at the bell stage of odontogenesis [58]. In OKC, SOX2 positivity was stronger and more diffuse in the intermediate epithelial layers [36,37,42], composed of cells with squamous differentiation, compared to the basal layer composed of cells with a preameloblast-like cellular phenotype [59]. Taken together, these findings agree with a theory based on the comparative transcriptomics analysis between OKC and AMBL [42], suggesting that OKC may develop from cells arrested at the dental lamina or bud stage, while AMBL progenitor cells may be more differentiated and may have reached the bell stage of odontogenesis [32].

The expression of OCT4 (Octamer-binding transcription factor 4), a member of the Pit-Oct-Unc (POU) family of transcription factors, encoded by the *POU5F1* gene, is evaluated in multiple studies (Table 1); however, divergent results were found, even between studies using anti-OCT4 antibodies from the same vendor [10,31]. However, further evaluation shows that four studies reporting no or limited OCT4 expression in benign tumors and developmental cysts used monoclonal antibodies [10,12,28,32], while one study showing OCT4 positivity applied a polyclonal antibody [31] and the relevant information was not available in another study [14]. In studies including both AMBL and OKC samples, the number of OCT4 positive cases was the same [31] or slightly higher in the OKC group [32], and our meta-analysis showed that OCT4 cannot be applied for distinguishing between OKC and AMBL. Interestingly, a study employing a monoclonal anti-OCT4 antibody found strong nuclear staining in the epithelial islands in 85% of ACA cases, while all AMBL samples were negative [12]. OCT4 has a vital role in the maintenance of self-renewal and pluripotency of ESCs [60], and regulates cell fate decisions by conducting an autonomous, but also synergistic, action with SOX2 [61]. OCT4 is another “Yamanaka factor”, crucial for the establishment of iPSCs [52], and has been implicated in the initiation and progression of several malignant tumors [62]. For example, oral cancer cells overexpressing the core pluripotency factors *SOX2* and *POU5F1*/OCT4 in xenograft mouse model assays were thought to represent reprogrammed cells capable of inducing tumorigenesis [63]. Whether the nuclear expression of OCT4 [12] and SOX2 [12,29,30] in the epithelial cells of ACA may be suggestive of their incomplete attempt for reprogramming, resulting in the loss of the typical features of ameloblastic differentiation and malignant transformation, is a hypothesis that merits further research. Further studies are required to evaluate whether OCT4 could distinguish between ACA and AMBL.

Of note, except for SOX2 and OCT4, the other two “Yamanaka factors” [52], i.e., KLF4 (Krüppel-like factor 4) and c-Myc, were also found to be expressed in some odontogenic lesions [37,45]. KLF4 is implicated in various cellular processes, including cell proliferation, apoptosis, and differentiation, and promotes terminal epidermal differentiation by inducing the expression of epithelial molecules and suppressing the expression of mesenchymal molecules [64,65]. RNA-seq analysis showed that the *KLF4* gene was upregulated in OKC compared to DF, and immunohistochemistry showed strong nuclear expression of the KLF4 protein, mainly in intermediate layers, and focally weak expression in the basal layer of OKC epithelia [37]. c-Myc is a proto-oncoprotein that acts as a transcription factor involved in cellular proliferation, apoptosis, and inhibition of differentiation [66]. Immunohistochemical expression of c-Myc was observed in the epithelial cells of most AMBL, AOT, and OKC cases, and in half or less than half of cases of RC and DC, respectively [45]. It is worth mentioning that among all odontogenic pathologies, OKC is the only one documented to express all four “Yamanaka factors” (Figure 2). As the endogenous expression of these factors has been linked to a higher intrinsic potential of somatic cells for cellular reprogramming [67,68], further research is warranted to elucidate whether cells isolated from OKC samples are amenable to reprogramming.

The expression of CD44, a surface glycoprotein encoded by the *CD44* gene, was also investigated in many studies (Table 1). CD44 acts as a core receptor for hyaluronic acid, regulating cell adhesion to extracellular matrix elements [69], and participates in the organization of a microenvironment conducive to the proliferation and stemness of tumor cells [70]. Strong membranous CD44 expression was observed in several odontogenic lesions, including AMBL, OKC, DC, and RC [11,12,31,44,46,48,50]. Interestingly, Marrelli et al. [43] showed that HRCMCs express elevated levels of CD44, as well as other stem cell markers, i.e., CD13, CD29, CD73, and CD90, similarly to DPSCs. In contrast, lower expression of CD105, a mesenchymal stem cell marker [20], was found in HRCMCs than DPSCs [43], as well as in RC tissue samples compared to periapical abscesses [11]. As RC is the most common odontogenic cyst encountered in oral pathology diagnostic services [4] and RC-derived cells can be isolated more easily than DPSCs after RC excision without additional surgical procedures, RC may represent a valuable cell source for regenerative dentistry [43,71].

Each one of the other fifteen stem cell markers included in the present analysis was evaluated in just one or two studies that usually included different odontogenic tumors and cysts (Table 1); thus, conclusions regarding their expression should be made with caution. Indications may indirectly arise, though, from studies evaluating different stem cell markers in the same odontogenic lesion. For example, a more intense expression of various stem cell markers, e.g., ALDH1 [34], CD166 [41], KLF4 [37], and SOX2 [37,42], was noted in the basal and, predominantly, the intermediate layers of OKC. These findings are indicative of inherent stem-cell-like properties [32] and are in line with the higher expression of proliferation markers in the same layers of the OKC epithelial lining [25], which may account for its locally aggressive behavior [72]. Most studies reported the expression of stem cell gene markers in tumor parenchyma or the cystic epithelial lining. However, different studies observed that some markers were expressed by both epithelial and mesenchymal cells, e.g., NANOG in AMBL and DC [10,14] or SOX2 in OKC [10,36,37,42], an observation that may be associated with the crucial role of stem cells in guiding reciprocal epithelial–mesenchymal interactions during the development of ectoderm-derived odontogenic tissues [5]. Only a few studies compared the expression of some stem cell markers between odontogenic lesions and control samples (Table 1); predominantly, SOX2 expression was compared between AMBL and DF in multiple studies with divergent results [12,14,32,38,40]. Thus, as confirmed by our meta-analysis (Figure S1), the expression of this marker does not seem to differ significantly between AMBL and DF. Similarly, no significant differences were noted in stem cell markers’ expression between RC and PG groups [11,50], except for a single study that found significantly lower ALDH1 expression in RC than PG [35].

Of note, three out of four studies included presented a moderate (unclear) or high RoB, predominantly due to the omission of information related to the diagnosis and clinical characteristics of the lesions or patients’ demographics. High risk of bias for the aforementioned items was also found in our previous systematic review [25], emphasizing the importance of following published guidance on reporting aspects about study participants’ enrollment, application and evaluation of methods, and outcome, depending on the study type [24]. In agreement with our previous work [25], the tendency for lower RoB in the most recent studies, i.e., during the last five years, was confirmed, indicating a trend or tendency for improved research quality.

A strength of the present systematic review is the inclusion of studies on human tissues, regardless of the fixation/processing method or the technique of stem cell marker evaluation, therefore resulting in more eligible studies. In addition, a thorough description of methodological details, as well as expression findings of the eligible studies is provided as supplementary material that may be useful in the design of future studies on stem cells’ expression. On the other hand, the fact that most stem cell markers were assessed only by one or two studies, as well as the small number of studies that investigated the expression of the same markers in the same odontogenic entities, may be regarded as study limitations.

## 5. Conclusions

The present study reviewed systematically for the first time the pertinent literature on stem cell gene expression in odontogenic tumors and cysts. It is suggested that stem cells may be linked to the development and clinical behavior of odontogenic pathologies and represent a potential target for future individualized therapeutic approaches. The outstanding discriminative ability of SOX2 for OKC vs. AMBL, as indicated by our meta-analysis, may be associated with their origin from cell populations at distinct stages of odontogenesis. Finally, our meta-analysis highlighted the significance of the SOX2 marker in the differential diagnosis of ACA vs. AMBL, which is of diagnostic value and should be verified by further studies.

## Figures and Tables

**Table 1:** Number (N) of cases in each odontogenic lesion/control group (Population), stem cell markers (Comparators), and results of the expression analysis (Outcome) in the 29 studies included in the qualitative synthesis.

Reference	Odontogenic Lesions; Controls (N)	Method	Marker *	Outcome of Comparison
Martins Balbinot et al., 2023 [14]	AMBL (23), DC (10); DF (10)	IHC	**SOX2**, **OCT4**, **NANOG**	Significantly higher scores of SOX2, OCT4, and NANOG were found in AMBL than DC (*p* < 0.001) and DF (*p* < 0.001).
da Trindade et al., 2022 [34]	AMBL (20), AOT (20), OKC (20), DC (20)	IHC	**ALDH1**	DC/OKC showed a significantly higher ALDH1 score than AMBL/AOT (*p* < 0.0001). No differences were found between the two cyst groups or between the two tumor groups.
Farias et al., 2022 [35]	RC (26); PG (25)	IHC	**ALDH1**	A lower ALDH1 immunoexpression score was found in RC than PG.
Júnior et al., 2022 [36]	OKC (20), BOC (10), GOC (10)	IHC	SOX2	SOX2 was expressed in OKC, while not in BOC or GOC.
Kalogirou et al., 2022 [37]	OKC (12); DF (6)	RNA-seq, IHC	**SOX2**, **KLF4**	SOX2 and KLF4 were upregulated in OKC compared to DF (*p* < 0.000). Higher immunoscores of SOX2 and KLF4 were found in OKC compared with DF.
Tseng et al., 2022 [38]	AMBL (49), UNAMBL (25); DF (6)	IHC	SOX2	DF showed a higher SOX2 score than all AMBL types.
Phattarataratip et al., 2021 [32]	AMBL (15), AOT (5), AF (5), OKC (15), DC (10), COC (5); DF (2)	IHC	**SOX2**, OCT4	A significantly higher SOX2 score was found in OKC compared with DC and AMBL (*p* < 0.001), and in AF compared with COC and AOT (*p* < 0.001). No difference was found in OCT4 score.
Chacham et al., 2020 [10]	AMBL (10), UNAMBL (10), OMYX (10), OKC (27), DC (10)	IHC	**NANOG**, **SOX2**, CD34, OCT4	A significantly higher NANOG immunoexpression score was found in DC/pOKC/UNAMBL than the other groups (except for pOKC vs. sOKC) (*p* < 0.05). A significantly higher SOX2 immunoscore was found in odontogenic tumors than in odontogenic cysts (except for UNAMBL vs. DC) (*p* < 0.05). A significantly higher CD34 immunoscore was found in rOKC than DC, and in pOKC/rOKC than UNAMBL/OMYX (*p* < 0.05). OCT4 was only expressed in a limited number of pOKC cells.
Silva et al., 2020 [33]	AMBL (20), OKC (20)	IHC	**SOX2**	A significantly higher SOX2 score was found in OKC than AMBL (*p* < 0.001).
Chang et al., 2020 [39]	AMBL (15), DC (6)	IF	**LGR5**	A significantly higher staining score of LGR5 was found in AMBL than DC (*p* < 0.0001).
Estrela et al., 2019 [11]	RC (10); PG (10), PAbs (10), APap (10)	IHC	**CD44**, **CD73**, **CD105**	Significantly higher expressions of CD44, CD73, and CD105 were found in the PAbs than RC/PG (*p* < 0.05).
Fraser et al., 2019 [40]	AMBL (5); DF (5)	IF	SOX2, BMI1	No difference was found.
Khan et al., 2018 [12]	AMBL (20), ACA (20); OSCC (5), DF (5)	IHC	**SOX2**, **OCT4**, CD44	Significantly higher SOX2 and OCT4 expressions were found in ACA than AMBL (*p* < 0.001).
Monroy et al., 2018 [31]	AMBL (20), AOT (20), OKC (20)	IHC	OCT4, **CD44**	A significantly higher CD44 score was found in OKC compared with AMBL and AOT (*p* = 0.034). No difference was found in OCT4 score.
Sanjai et al., 2018 [30]	AMBL (11), ODAMBL (2), ACA (6)	IHC	SOX2	No difference was found.
Bandyopadhyay et al., 2017 [28]	AMBL (15), OKC (15)	IHC	SOX2, OCT4	SOX2 was expressed only in OKC. No difference in OCT4 score.
Andisheh-Tadbir & Gorgizadeh, 2016 [41]	AMBL (17), UNAMBL (15), OKC (18), DC (19)	IHC	**CD166**	A significantly higher CD166 expression was found in AMBL/UNAMBL/OKC than DC (*p* < 0.005).
Heikinheimo et al., 2015 [42]	AMBL (15), OKC (12); NOM (4)	microarrays, real-time RT-PCR, IF	**SOX2**	SOX2 was upregulated in OKC compared to AMBL (*p* < 0.000). SOX2 immunoexpression was found only in OKC.
Marrelli et al., 2015 [43]	HRCMCs (4); DPSCs (4)	flow cytometry, real-time RT-PCR	CD13, CD29, CD44, CD73, CD90, CD105	Lower CD105 expression was found in HRCMSs than DPSCs. No other differences were found.
Lei et al., 2014 [29]	AMBL (16), IGaAMBL (6), ACA (13)	IHC	**SOX2**	A significantly higher SOX2 score was found in ACA than AMBL/IGaAMBL (*p* = 0.0021).
Srinath et al., 2014 [44]	AMBL (10), DC (5) RC (5)	IHC	CD44	No difference was found.
Juuri et al., 2013 [6]	AMBL (5); HPMDE (NA)	IHC	SOX2	SOX2 was expressed in AMBL and dental lamina.
Moosvi & Rekha, 2013 [45]	AMBL (10), AOT (10), OKC (10), DC (10), RC (10)	IHC	**c-Myc**	A significantly higher intensity score was found in OKC than DC (*p* = 0.0257) and RC (*p* = 0.0452); in AMBL than DC (*p* = 0.0312); and in AOT than DC (*p* = 0.0257) and RC (*p* = 0.0452). No differences between OKC, AMBL, and AOT.
Salehinejad et al., 2012 [46]	OKC (14), DC (14), RC (14), COC (14)	IHC	**CD44v6**	A significantly higher CD44v6 score was found in OKC compared with DC (*p* < 0.001), RC (*p* < 0.001), and COC (*p* = 0.024), and in COC compared with DC (*p* < 0.001) and RC (*p* < 0.001).
Kumamoto et al., 2010 [13]	AMBL (47), metAMBL (2), ACA (2), CCOCa (2); TG (12)	IHC, RT-PCR	**CD133**, BMI1, **ABCG2**	A significantly higher ABCG2 score was found in AMBL/metAMBL/ACA than TG (*p* < 0.05), and a significantly higher CD133 score was found in malAMBL/ACA than AMBL (*p* < 0.001) and TG (*p* < 0.001). No difference was found in BMI1 score.
Song et al., 2009 [47]	HODCs; DPSCs, BMSCs	IF, real-time RT-PCR	**Nestin**, SOX2	A significantly higher expression level of Nestin mRNA was found in HODCs compared with DPSCs/BMSCs (*p* < 0.04). SOX2 was expressed only in HODCs.
Wang & Liu, 2009 [48]	OKC (20), DC (8), RC (10)	IHC	CD44v6	No difference was found.
Fujita et al., 2006 [49]	AMBL (44), malAMBL (3), ODO (62), AF (2), AFD (7), AFSa (2), AOT (6), OdF (3)	IHC	Nestin	Nestin was expressed in odontogenic mixed tumors, mainly in sites of odontogenic ectomesenchyme.
Leonardi et al., 2000 [50]	RC (4); PG (16)	IHC	CD44	No difference was found.

Abbreviations: AMBL, ameloblastoma; ACA, ameloblastic carcinoma; AF, ameloblastic fibroma; AFD, ameloblastic fibro-odontoma/fibrodentinoma; AFSa, ameloblastic fibrosarcoma; AOT, adenomatoid odontogenic tumor; APap, apical papillae; BMSCs, bone marrow stromal cells; BOC, botryoid odontogenic cyst; CCOCa, clear cell odontogenic carcinoma; COC, calcifying odontogenic cyst; DC, dentigerous cyst; DF, dental follicle; DPSCs, dental pulp stem cells; GOC, glandular odontogenic cyst; HODCs, human odontoma-derived mesenchymal cells; HPMDE, human primary molar dental epithelium; HRCCs, human RC-derived cells; IF, immunofluorescence; IGaAMBL, intermediate-grade atypical AMBL; IHC, immunohistochemistry; malAMBL, malignant AMBL; metAMBL, metastasizing AMBL; NA, not available; NOM, normal oral mucosa; ODAMBL, odontoameloblastoma; OdF, odontogenic fibroma; ODO, odontoma; OKC, odontogenic keratocyst; OMYX, odontogenic myxoma; OSCC, oral squamous cell carcinoma; PAbs, periapical abscess; PG, peripheral granuloma; pOKC, primary odontogenic keratocyst; RC, radicular cyst; RNA-seq, RNA-sequencing; rOKC, recurrent odontogenic keratocyst; RT-PCR, reverse transcription-polymerase chain reaction; sOKC, syndromic odontogenic keratocyst; TG, tooth germ; UNAMBL, unicystic ameloblastoma. * Markers found to be differentially expressed between different odontogenic lesions or between an odontogenic tumor/cyst group and a control group by statistical analysis are marked as bold.

## Data Availability

The original contributions of the study are included in the article and Supplementary Materials. Further inquiries can be directed to the corresponding author.

## Supplementary Materials

The following supporting information can be downloaded at: https://www.mdpi.com/article/10.3390/genes14091735/s1, Table S1. Keywords applied for literature search in each database. Table S2. Records excluded from qualitative analysis and reasons for exclusion at specific search phases. Table S3. Journal of publication, disease and control groups, sample type, number of patients, demographic characteristics, site of samples, documentation of disease/control entities’ diagnosis, and histopathologic variants in case of solid ameloblastomas. Table S4. Stem cell markers used in immunohistochemical and immunofluorescence analysis. Table S5. Stem cell markers used in studies applying flow-cytometry, PCR, microarray, or RNA-seq methods. Table S6. Outcome of comparison of stem cell markers between odontogenic lesions with immunohistochemical and immunofluorescence analysis. Table S7. Outcome of comparison of stem cell markers between odontogenic lesions with flow-cytometry, PCR, microarray, or RNA-seq methods. Table S8. Interpretation of the Critical Appraisal Tool proposed by the Joanna Briggs Institute for risk of bias assessment in the present study. Table S9. Risk of bias assessment using the Critical Appraisal Tool proposed by the Joanna Briggs Institute. Table S10. Eight studies included in the meta-analysis. Figure S1. Forest plots of the pooled Sensitivity (A), Specificity (B), LR+ (C), LR- (D), and DOR (E) of the two studies involving the SOX2 biomarker for the AMBL and DF disease pair. Since there are only two studies, a ROC plot (F) was produced instead of an SROC curve, and as such, AUC was not calculated. Figure S2. Forest plots of the pooled Sensitivity (A), Specificity (B), LR+ (C), LR- (D), and DOR (E) showing the two studies involving the POU5F1 biomarker for the OKC and AMBL disease pair. Since there are only two studies, a ROC plot (F) was produced instead of an SROC curve, and as such, AUC was not calculated.
