# Supplementary figures and images for: The Stem Cell Expression Profile of Odontogenic Tumors and Cysts: A Systematic Review and Meta-Analysis
Source: Genes (Basel). 2023 Aug 30;14(9):1735. doi: 10.3390/genes14091735 (PMC10531260; doi:10.3390/genes14091735)

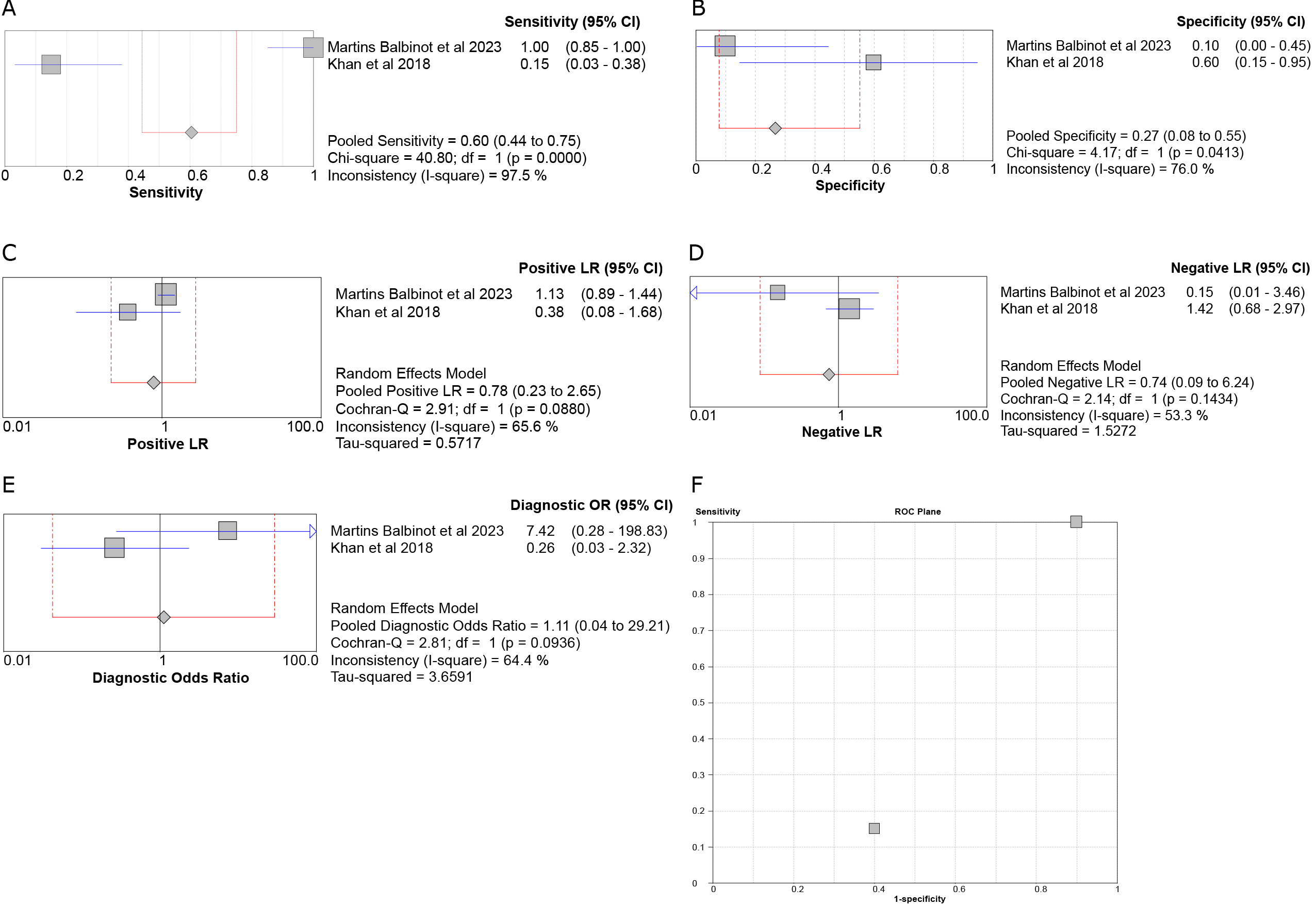

Supplement: Supplementary file 1 [file genes-14-01735-s001.zip › Figure S1.tif]

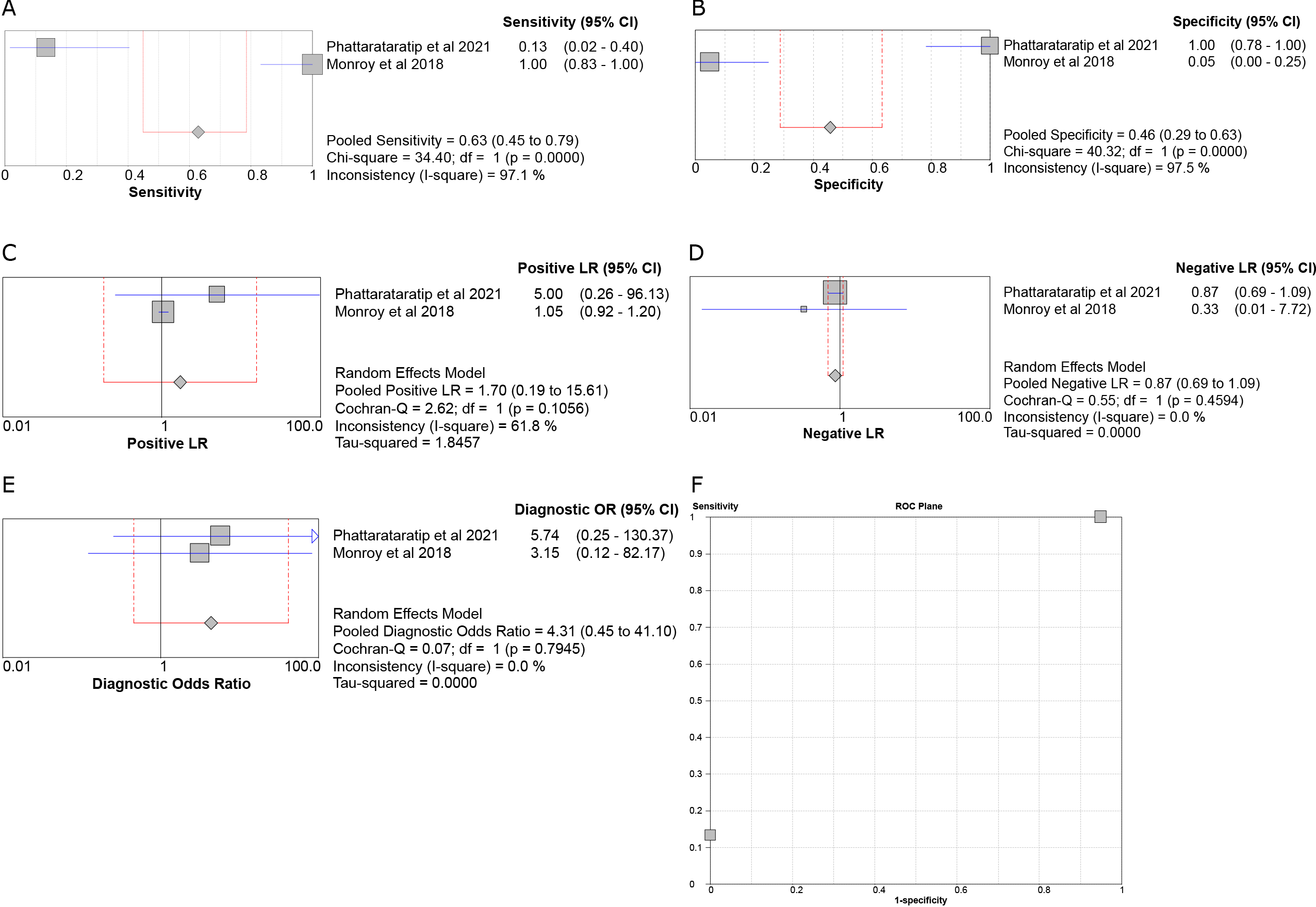

Supplement: Supplementary file 1 [file genes-14-01735-s001.zip › Figure S2.tif]
